# Hepatitis B virus X protein binding to hepsin promotes C3 production by inducing IL-6 secretion from hepatocytes

**DOI:** 10.18632/oncotarget.6846

**Published:** 2016-01-08

**Authors:** Mingming Zhang, Jianxin Gu, Chunyi Zhang

**Affiliations:** ^1^ Department of Biochemistry and Molecular Biology, Gene Research Center, School of Basic Medical Sciences, Fudan University, Shanghai 200032, China

**Keywords:** hepatitis B virus X protein, hepsin, complement C3, interleukin-6, hepatocellular carcinoma

## Abstract

Hepatitis B virus (HBV) X protein (HBx) is an important effector for HBV-associated pathogenesis. In this study, we identified hepsin as an HBx-interacting protein and investigated the effects of hepsin on HBx-mediated complement component 3 (C3) secretion in hepatocytes. *In vivo* and *in vitro* binding between HBx and hepsin was confirmed by co-immunoprecipitation and Glutathione S-transferase pull-down assays. HBx synergized with hepsin to promote C3 production by potentiating interleukin-6 (IL-6) secretion. Knockdown of endogenous hepsin attenuated C3 and IL-6 secretion induced by HBx in hepatic cells. In addition, levels of hepsin protein correlated positively with C3 expression in human non-tumor liver tissues. Further exploration revealed that HBx and hepsin increased C3 promoter activity by up-regulating the expression and phosphorylation of the transcription factor CAAT/enhancer binding protein beta (C/EBP-β), which binds to the IL-6/IL-1 response element in the C3 promoter. HBx and hepsin synergistically enhanced IL-6 mRNA levels and promoter activity by increasing the nuclear translocation of nuclear factor kappaB (NF-κB). Our findings show for the first time that binding between HBx and hepsin promotes C3 production by inducing IL-6 secretion in hepatocytes.

## INTRODUCTION

As part of innate and acquired immune responses, the complement system is involved in the pathogenesis of a variety of liver disorders, including viral hepatitis, liver repair after injury, fibrosis and alcoholic liver disease [1]. Complement factors and other humoral factors are increased in response to viral infections [2–4]. Complement component 3 (C3), which plays a vital role in both the classical and alternative pathways of complement activation, is an acute-phase protein (APP) whose expression is regulated by interleukin-6 (IL-6), IL-1, tumor necrosis factor alpha (TNF-α) and glucocorticoids (GCs) [9]. IL-6 has been shown to induce hepatocyte proliferation and liver regeneration, and increase animal survival after partial hepatectomy [5]. C3 deposition was significantly up-regulated in virus-induced fulminant hepatitis (FH) patients [6]. Serum levels of circulating IgG and C4 are significantly lower in occult hepatitis B infection patients than in healthy controls, while IgM and C3 levels are higher [7]. Other reports have shown that serum C3 concentrations are reduced in acute viral hepatitis [8, 9]. These data suggest that whether C3 is increased or decreased is dependent on the type and stage of hepatitis virus B (HBV) infection.

Chronic HBV infection is a leading cause of cirrhosis and hepatocellular carcinoma (HCC). The role of the adaptive immune response in HBV infections is well established. HBV has a partially double-stranded DNA genome with four overlapping open reading frames (ORFs). The smallest ORF, hepatitis B virus X protein (HBx), encodes a 17-kDa soluble protein that is expressed at low levels during acute and chronic hepatitis, and induces a humoral and cellular immune response [10–13]. HBx appears to promote carcinogenesis by enhancing cell proliferation, inhibiting cell apoptosis, and increasing cell invasive potential. HBx also can induce long non-coding RNA DBH-AS1, and consequently promotes cell proliferation and survival in HCC by activating MAPK signaling [14]. HBx-induced alpha-fetoprotein (AFP) expression promotes malignant transformation in liver cells through activation of PI3K/mTOR signaling [15]. Finally, HBx shifts the oncogenic addiction of HCC cells to the ErbB2/ErbB3 signaling pathway and enhances their sensitivity to the EGFR/ErbB2 inhibitor lapatinib by up-regulating ErbB3 expression [16].

Hepsin, a type II transmembrane serine protease [17], is expressed in most tissues, with its highest levels in liver. *In vitro* studies suggest hepsin is required for the growth and maintenance of normal morphology in human hepatocytes [18], as well as for cell motility [19] and development [20], initiation of blood coagulation [21] and proinflammatory immune responses [22].

In this study, we show that HBx promotes C3 secretion from hepatic cells. We identify hepsin as a signaling molecule that interacts with HBx to increase C3 production by selectively inducing IL-6 secretion in hepatocytes, which provides a mechanism for the control of HBV-specific cellular immune responses.

## RESULTS

### The association between HBx and hepsin *in vivo* and *in vitro*

**Figure 1:**
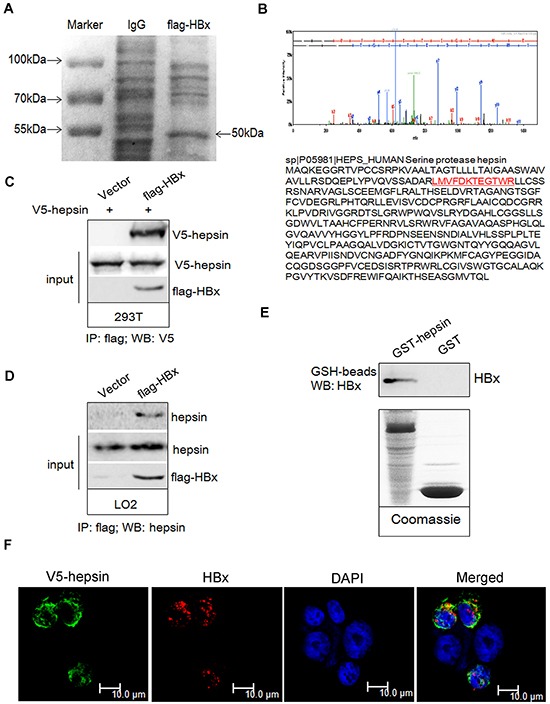
HBx binds to hepsin *in vivo* and *in vitro* Immunoprecipitation was performed as described. **A.** Coomassie blue staining showed the appearance of a ~50 kDa band in the lysates of flag-HBx transfected 293T cells. **B.** The ~50 kDa band was subjected to mass spectrometric analysis and identified as a unique peptide of hepsin. **C.** 293T cells were transiently transfected with V5-hepsin or combinations of flag-HBx, respectively. The lysates were immunoprecipitated with an anti-flag antibody, followed by western blot analysis with an anti-V5 antibody. 10% whole cell lysate (input) was probed for the expression of hepsin and HBx. **D.** LO2 cells were transiently transfected with empty or flag-HBx vector. The lysates were immunoprecipitated with an anti-flag antibody, followed by western blot analysis with an anti-hepsin antibody. 10% whole cell lysate (input) was probed for the expression of hepsin and HBx. **E.** GST-hepsin or GST bound to glutathione-Sepharose beads were incubated with purified recombinant HBx protein. Precipitates were subjected to SDS-PAGE and western blot analysis with anti-HBx antibody. The lower panel shows coomassie staining of purified GST-hepsin and GST proteins. **F.** Confocal microscopy revealed co-localization of HBx and hepsin in LO2 cells. Cells were transfected with V5-hepsin and flag-HBx plasmids, and fixed; 48 hours later, confocal immunofluorescence microscopy assay was performed. HBx (red), hepsin (green) and DAPI (blue) are shown. Colocalization of the two fluorescent dyes produced a yellow color. Scale bars = 10 μm.

First, immunoprecipitates obtained with anti-flag antibodies were resolved on an SDS-polyacrylamide gel. Coomassie Blue staining of the gel revealed a 50 kDa band in flag-HBx transfected 293T cells (Figure 1A). Mass spectrometry was used for identification of hepsin in flag-HBx immunoprecipitates (Figure 1B). Next, we confirmed the binding between HBx and hepsin. Upon expression in 293T cells, HBx and hepsin formed a physical complex (Figure 1C). Using an anti-flag antibody for immunoprecipitation and an anti-hepsin antibody for immunoblotting, we also found that endogenous hepsin bound to HBx in human immortalized hepatic LO2 cells (Figure 1D). To test whether HBx bound directly to hepsin, we performed Glutathione S-transferase (GST) pull-down assays *in vitro*. Purified recombinant HBx protein was precipitated by GST-hepsin, but not by GST alone (Figure 1E). To determine whether hepsin was co-localized with the HBx protein in human hepatocytes, we co-transfected plasmids encoding V5-hepsin and flag-HBx proteins into LO2 cells. Hepsin was mainly localized in the cytoplasm and the cell membrane, while HBx was localized predominantly in the cytoplasm of the LO2 cells. As hepsin is a transmembrane serine protease expressed on the cell surface, merged images showed hepsin and HBx colocalization in the cytoplasmic region adjacent to the cell membrane in LO2 cells (Figure 1F).

### HBx binding to hepsin induces C3 production in hepatocytes

**Figure 2:**
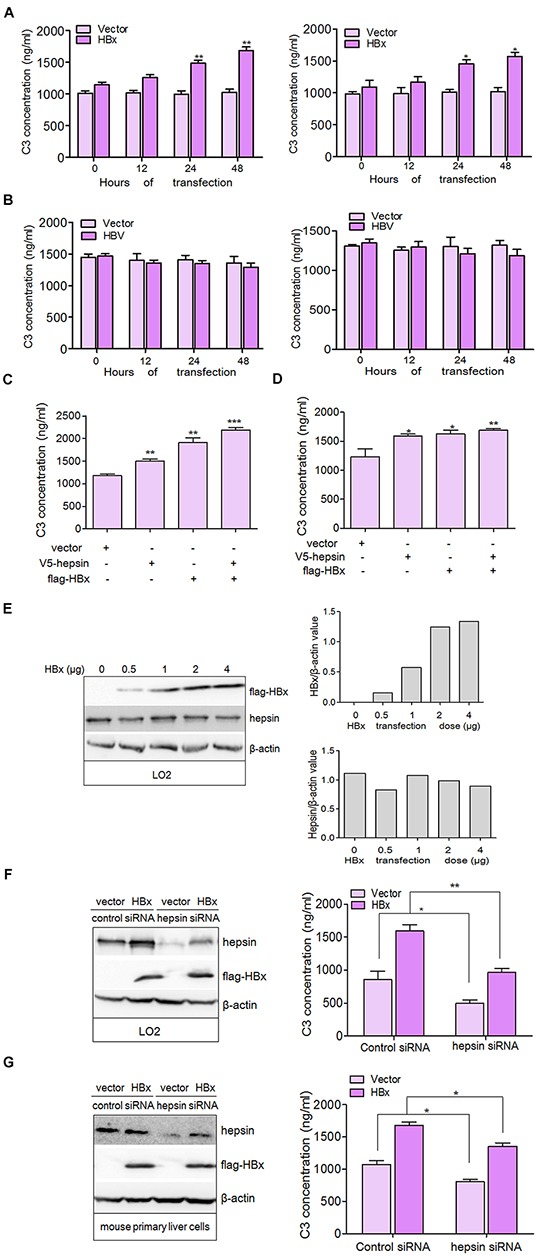
HBx binding to hepsin induces C3 production in human hepatocytes **A.** LO2 (left panel) and mouse primary liver cells (right panel) were transfected with flag-HBx or empty vector and C3 levels in the culture medium were measured by ELISA. Data are presented as mean ±SEM of three independent experiments. *P < 0.05, **P < 0.01. **B.** LO2 (left panel) and mouse primary liver cells (right panel) were transfected with HBV or empty vector and C3 levels in the culture medium were measured by ELISA. **C.** and **D.** Expression of HBx and hepsin in hepatocytes increased the production of C3. LO2 and mouse primary liver cells were transfected with V5- hepsin and flag-HBx alone or together. C3 secretion was measured by ELISA. Data are presented as mean ±SEM of triplicate experiments. *P < 0.05, **P < 0.01, ***P < 0.001. **E.** HBx does not change the expression of hepsin. LO2 cells were transfected with increasing amounts of HBx expression vector. The extracted lysates were analyzed by western blotting (left panel) with antibodies against flag, hepsin or β-actin. The expression of flag-HBx (upper panel) and hepsin (lower panel) was quantitated against β-actin. **F.** and **G.** C3 secretion is correlated with the normal expression of hepsin. LO2 and mouse primary liver cells transfected by control siRNA or hepsin siRNA were co-transfected with or without flag-HBx, and C3 secretion was measured by ELISA. Data are presented as mean ±SEM. Knockdown efficiency of hepsin, and the expression of HBx was examined by immunoblotting. β-actin was used as a loading control. *P < 0.05, **P < 0.01.

To investigate the role of HBx and HBV on C3 production in parenchymal liver cells, we detected C3 secretion using enzyme-linked immunosorbent assay (ELISA) in transfected hepatic cells. C3 levels in the culture medium in empty vector-transfected cells remained steady, while expression of flag-HBx dramatically enhanced C3 secretion in a time-dependent manner both in LO2 and mouse primary liver cells (Figure 2A). C3 secretion appeared to decrease in HBV-transfected hepatic cells, although this was not statistically significant (Figure 2B). Immunoblot analysis with HBV-transfected LO2 cells confirmed the expression of core protein (Supplementary Figure S1). A weak reduction in C3 secretion was observed in HBc transfected LO2 cells compared to empty vector-transfected cells (Supplementary Figure S2).

Because hepsin associated with HBx in LO2 cells, we examined the combined effect of HBx and hepsin on C3 production. Expression of HBx or hepsin alone promoted C3 secretion more than cells transfected with empty vector (Figure 2C and 2D). Co-expression of both hepsin and HBx resulted in stronger secretion of C3 than either hepsin or HBx expression alone, indicating a cooperative effect between hepsin and HBx. Additionally, we found that expression of endogenous hepsin was not affected by transfection with increasing amounts of HBx (Figure 2E). These results suggest that the cooperation between HBx and hepsin is not mediated by a change in hepsin levels, but by the possible interaction between HBx and hepsin. Further studies revealed that knockdown of hepsin dampened C3 production with or without HBx transfection (Figure 2F and 2G), and showed that HBx increased C3 production by binding to hepsin in hepatocytes.

### HBx synergizes with hepsin to enhance C3 promoter activation

**Figure 3:**
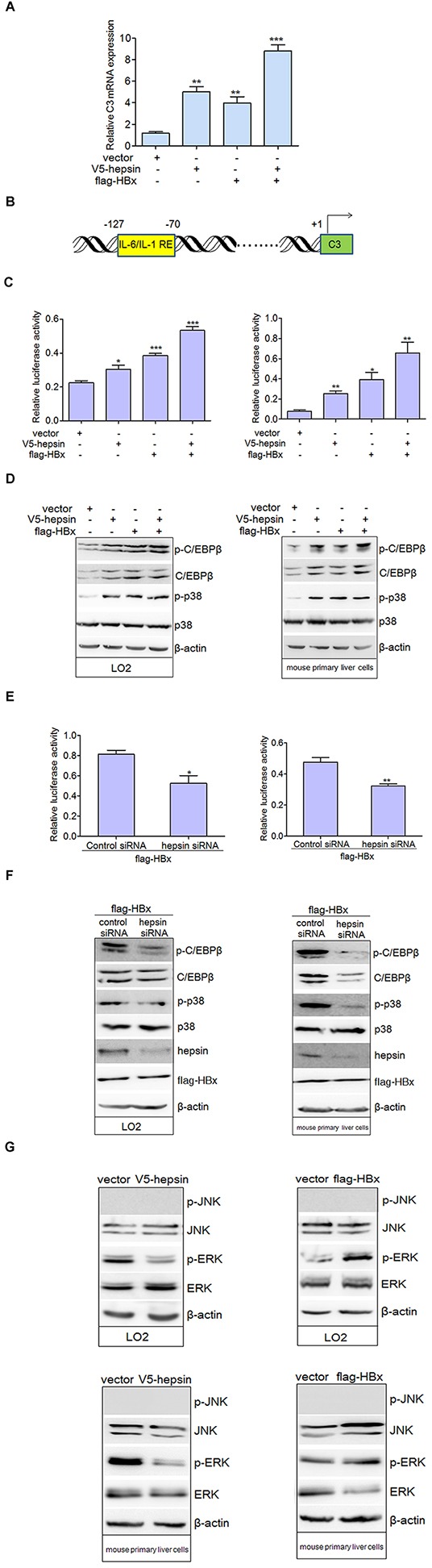
HBx synergizes with hepsin to promote C3 promoter activation **A.** HBx and hepsin in human hepatocytes synergistically increased mRNA expression of C3. LO2 cells were transfected with empty vector, expression vectors for V5-hepsin, flag-HBx, or both V5-hepsin and flag-HBx. C3 mRNA was detected by real-time PCR. Error bars indicate SEM. Results were normalized to endogenous GAPDH. **P < 0.01, ***P < 0.001. **B.** Schematic diagram of the human C3 promoter containing IL-6/IL-1 REs for reporter gene assay. **C.** C3 promoter activity was induced by HBx and hepsin. LO2 (left panel) and mouse primary liver cells (right panel) were transfected, and relative IRES activities were detected. *P < 0.05, **P < 0.01, ***P < 0.001. **D.** LO2 and mouse primary liver cells were transfected with expression vectors as indicated. Phosphorylated and total levels of p38 MAPK and C/EBP-β were detected. Cellular actin was used as a protein loading control in each lane. **E.** and **F.** Knockdown of hepsin in hepatocytes attenuated C3 promoter activation and the phosphorylation levels of p38 MAPK and C/EBP-β induced by HBx. *P < 0.05, **P < 0.01. **G.** Total and phosphorylated ERK and JNK were measured by western blot in transfected hepatic cells.

We evaluated the effect of hepsin and HBx on C3 mRNA expression in hepatocytes, and found that both hepsin and HBx increased C3 mRNA expression (Figure 3A). The co-expression of hepsin and HBx resulted in even stronger C3 up-regulation than either HBx or hepsin alone. To study the impacts of HBx and hepsin on C3 promoter activity, a C3 promoter construct with a luciferase reporter gene (Figure 3B) was transfected into LO2 and mouse primary liver cells along with plasmids encoding hepsin and HBx. Cells were lysed 48 hours after transfection, and promoter activity was measured by luciferase assay. Co-transfection of HBx and hepsin increased C3 promoter activity more than either hepsin or HBx expression alone (Figure 3C).

C/EBP-β, a member of the CCAAT/enhancer binding protein family, is one of the key transcription factors responsible for induction of various APP genes including C3 [23–25]. C/EBP-β can be phosphorylated by activated p38 MAPK, which is essential for its DNA-binding ability and transcriptional activity [26, 27]. Our results suggested that the expression of total and phosphorylated C/EBP-β, as well as phospho-p38 MAPK levels, were up-regulated in hepatocytes transfected with hepsin, HBx or both plasmids together (Figure 3D). Knockdown of hepsin in HBx-transfected cells repressed C3 promoter activity (Figure 3E) and decreased phosphorylation of p38 MAPK and C/EBP-β in two hepatic cell lines (Figure 3F). These data indicate that the p38 MAPK-C/EBP-β pathway is involved in C3 expression induced by HBx. In addition to p38 MAPK, C/EBP-β expression can be induced by JNK or ERK activation [28]. Our results showed that expression of HBx increased phosphorylation of ERK, but not JNK. On the contrary, hepsin expression led to down-regulation of phospho-ERK in LO2 and mouse primary liver cells (Figure 3G).

### IL-6 is the major activator of C3 production in hepatocytes

**Figure 4:**
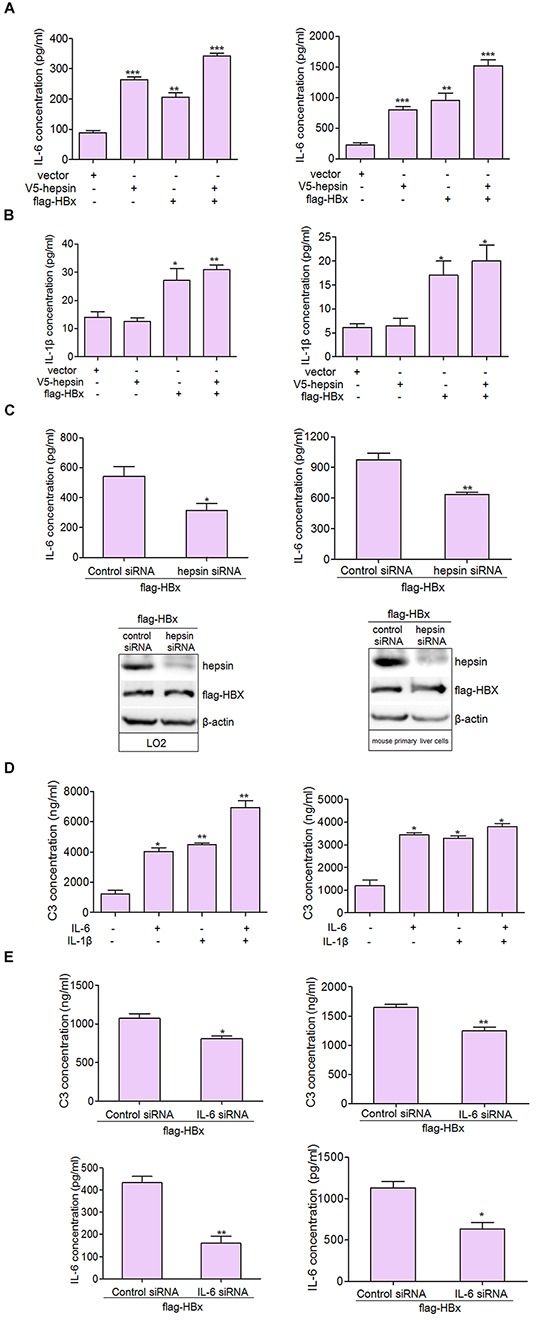
IL-6 is the major activator of C3 production in hepatocytes **A.** HBx and hepsin in LO2 and mouse primary liver cells synergistically increased IL-6 secretion. LO2 and mouse primary liver cells were transfected with hepsin and HBx alone or together, and IL-6 secretion was measured by ELISA. Data are presented as mean ±SEM of three independent experiments. **P < 0.01, ***P < 0.001. **B.** Cells were transfected with expression vectors as indicated, and culture supernatants were collected, centrifuged and subjected to IL-1β ELISA. Data shown represent the mean ±SEM of three independent experiments. *P < 0.05, **P < 0.01. **C.** Knockdown of hepsin decreased HBx induced IL-6 production. **D.** IL-6 and IL-1β up-regulated secretion of C3. LO2 and mouse primary liver cells were treated with IL-6, and IL-1β alone or togeter. C3 secretion was measured by ELISA. Data are presented as mean ±SEM of triplicate experiments. *P < 0.05, **P < 0.01. **E.** Knockdown of IL-6 in LO2 cells (left panel) and mouse primary liver cells (right panel) suppressed HBx induced C3 secretion. *P < 0.05, **P < 0.01.

As with other APPs, the expression of C3 is regulated by IL-6, IL-1, TNF-α, and GCs [29]. IL-6 is a pleiotropic cytokine that is important in the induction of immune reactions and inflammatory responses. In the liver, IL-6 regulates the synthesis of a broad spectrum of APPs. Our results suggest that IL-6 secretion could be induced by HBx and hepsin together in LO2 and mouse primary liver cells (Figure 4A). IL-1β, another pro-inflammatory cytokine, was only weakly up-regulated from low basal levels upon HBx overexpression (Figure 4B). We did not detect the secretion of TNF-α in basal or transfected conditions (data not shown). Further studies revealed that knockdown of hepsin suppressed IL-6 production induced by HBx in hepatic cells (Figure 4C).

We then examined C3 secretion in hepatocytes following treatment with IL-6 and IL-1β. Our findings were consistent with a previous report [30], and suggested that IL-6 and IL-1β alone or together enhanced C3 secretion (Figure 4D). In hepatic cells, IL-6 knockdown inhibited C3 production induced by HBx (Figure 4E). These results support IL-6 as a likely activator for C3 production in hepatocytes.

### HBx and hepsin enhance IL-6 expression via the nuclear translocation of NF-κB

**Figure 5:**
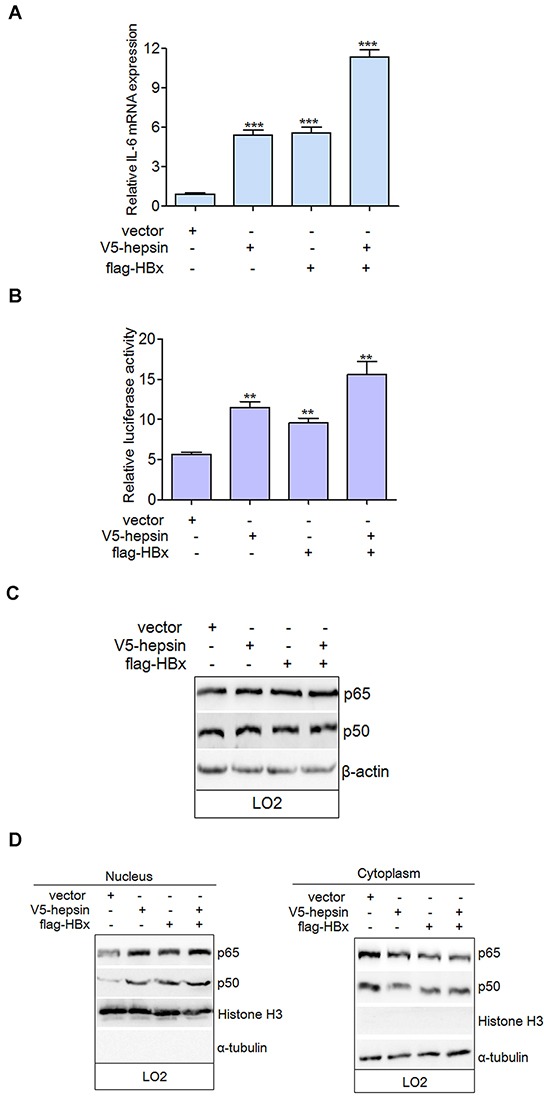
HBx synergizes with hepsin to increase IL-6 mRNA level and promoter activation by stimulating the nuclear translocation of NF-κB **A.** HBx and hepsin in human hepatocytes synergistically increased mRNA expression of IL-6. LO2 cells were transfected with empty vector, expression vectors for V5-hepsin, flag-HBx, or both. IL-6 mRNA was detected by real-time PCR. Error bars indicate SEM. Results were normalized to endogenous GAPDH. ***P < 0.001. **B.** IL-6 promoter activity was induced by HBx and hepsin. LO2 cells were transfected and relative IRES activities were detected. **P < 0.01. **C.** LO2 cells were transfected with expression vectors as indicated. Cell extracts were prepared and analyzed by western blot using anti-p65, anti-p50, and anti-β-actin antibodies. **D.** Cytoplasmic and nuclear fractions of transfected LO2 cells were prepared as described. Both cytoplasmic and nuclear proteins were analyzed with anti-p65 and anti-p50 antibodies to determine the localization of NF-κB subunits. An antibody against nuclear-specific histone H3 and an antibody against cytoplasmic-specific α-tublin were used as controls.

Real-time PCR and luciferase activity analyses revealed that hepsin or HBx could up-regulate the mRNA expression and promoter activity of IL-6 (Figure 5A and 5B). Co-expression of hepsin and HBx resulted in even stronger IL-6 up-regulation than either HBx or hepsin alone. IL-6 is a NF-κB-mediated gene, and NF-κB can bind directly to its promoter and increase its expression. However, NF-κB subunit p65 and p50 levels remained the same with or without transfection, suggesting that hepsin and HBx did not alter NF-κB expression (Figure 5C). To determine the localization of NF-κB, cytoplasmic and nuclear fractions were prepared and detected using the same antibodies as for hepsin and HBx. The nuclear-specific antibody histone H3 and cytoplasm-specific antibody α-tublin were used as controls to ensure equal loading of the fractions and exclude the possibility of cross contamination. Hepsin and HBx promoted the translocation of p65 and p50 to the nucleus, thereby decreasing the amounts of cytoplasmic p65 and p50 (Figure 5D). These data demonstrate that HBx synergizes with hepsin to increase IL-6 mRNA levels and promoter activation by stimulating the nuclear translocation of NF-κB subunits.

### The IL-6 and p38 MAPK-C/EBP-β pathways regulate C3 promoter activity

**Figure 6:**
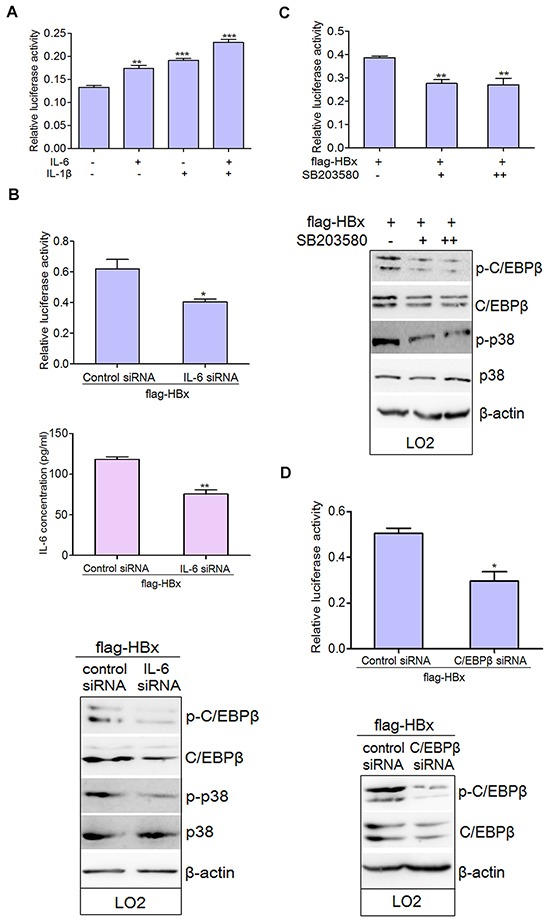
IL-6 and p38 MAPK-C/EBP-β pathway are involved in the regulation of C3 promoter activity in hepatocytes **A.** IL-6 and IL-1β elevated C3 promoter activity. LO2 cells were treated with IL-6 and IL-1β alone or together. The promoter activity of C3 was observed. Data are presented as mean ±SEM of triplicate experiments. **P < 0.01, ***P < 0.001. **B.** HBx-transfected LO2 cells were transfected with control siRNA or IL-6 siRNA for 72 hours. Protein lysates were prepared and the total and phosphorylated levels of p38 MAPK and C/EBP-β were determined by western blot analysis. Relative C3 promoter activity was detected. *P < 0.05, **P < 0.01. **C.** HBx-transfected LO2 cells were cultured without addition (−) or treated with 10μM (+) or 20μM (++) of SB203580 for 24 hours. Cells were lysed in order to assess luciferase activity and protein expression. **P < 0.01. **D.** HBx transfected LO2 cells were treated with control siRNA or C/EBP-β siRNA for 72 hours. Relative C3 promoter activity was detected. *P < 0.05.

Type I IL-6/IL-1 responsive elements (REs) have been identified on the promoters of most type I APP genes, including C3 [24, 25]. These are binding sites for C/EBP transcription factors [31], which can activate transcription induced by both IL-6 and IL-1. Consistent with this, treatment with IL-6 and IL-1β together increased C3 promoter activity (Figure 6A). As HBx and hepsin mainly stimulated IL-6 secretion in hepatocytes, silencing IL-6 via small interfering RNA (siRNA) dramatically down-regulated the promoter activity of C3 by inhibiting the phosphorylation of p38 MAPK and C/EBP-β in LO2 cells (Figure 6B). SB203580, a specific p38 MAPK inhibitor, inhibited C3 promoter activity as compared to HBx-transfected cells alone (Figure 6C). Moreover, SB203580 suppressed phosphorylation of p38 MAPK and C/EBP-β, but had no effect on total p38 MAPK expression (Figure 6C). The involvement of C/EBP-β as a transcription factor for C3 promoter activity in hepatocytes was verified by C/EBP-β knockdown (Figure 6D). Taken together, these experiments provide strong evidence for control of C3 promoter activity by IL-6 and the p38 MAPK-C/EBP-β pathway.

### C3 protein levels are correlated with hepsin expression in human non-tumor liver tissues

**Figure 7:**
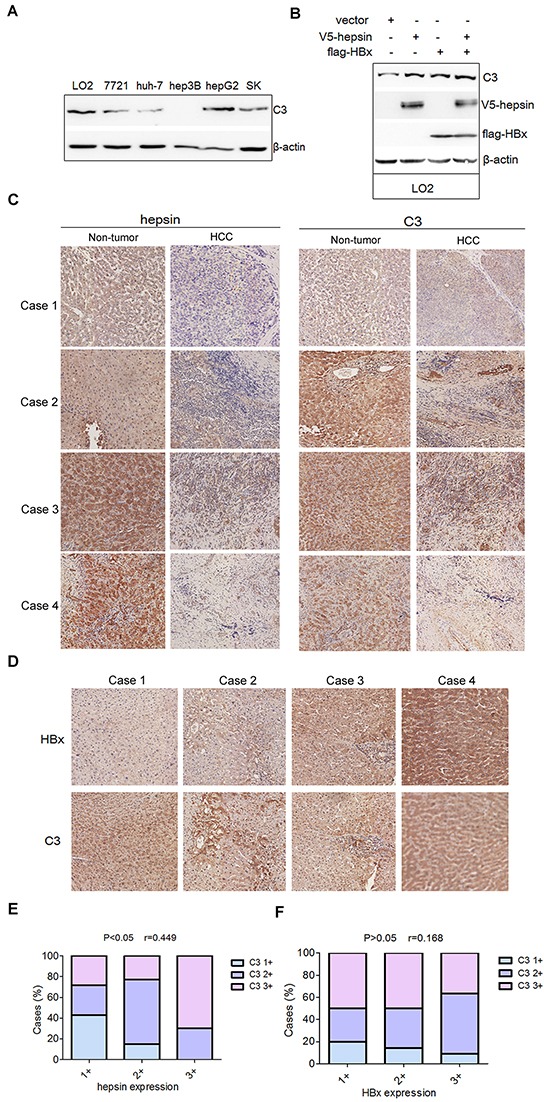
Levels of C3 protein are positively correlated with hepsin expression in human non-tumor liver tissues **A.** Proteins extracted from LO2 and different human HCC cell lines were subjected to western blot with C3 antibody. **B.** LO2 cells was transfected with V5-hepsin, or flag-HBx, or both. The expression of C3 was measured by western blot. **C.** Representative immunohistochemistry staining with different hepsin and C3 expression levels (20×) in 4 pairs of non-tumor liver tissues and HCC tissues. **D.** Representative immunohistochemistry staining with HBx and C3 (20×) in human HBV-infected liver tissues. **E.** Correlation analysis between hepsin and C3 expression in human non-tumor liver tissues (*P* < 0.05, *r* = 0.449, *n* = 30). **F.** Correlation analysis between HBx and C3 expression in human HBV-infected liver tissues (P>0.05, *r* = 0.168, *n* = 35).

Western blot analysis revealed that most of the human HCC cell lines (7721, hun-7, hepG2 and SK) and the human hepatocyte line LO2 exhibited C3 expression in lysates (Figure 7A). Moreover, in the LO2 cells, hepsin and HBx promoted C3 expression (Figure 7B). Previous study revealed that the expression levels of hepsin in non-tumor liver tissues are higher than those in the corresponding HCC tissues [32]. Consistent with this, our clinical HCC tissues exhibited decreased hepsin expression in comparison to matched non-tumor liver tissues (Figure 7C, Supplementary Table S1). However, C3 levels between HCC and matched non-tumor liver tissues were not significantly different (Figure 7C, Supplementary Table S2). As the expression of hepsin was very low in HCC tissues, correlation analysis based on immunohistochemical staining was studied in 30 non-tumor liver tissues. Correlation analysis between hepsin and C3 staining revealed that hepsin expression was positively correlated with C3 expression (*P* < 0.05, *r* = 0.449, *n* = 30) (Figure 7E, Supplementary Table S3). However, levels of HBx protein in clinical HBV-infected liver tissues were not correlated with C3 expression (P>0.05, *r* = 0.168, *n* = 35) (Figure 7D and 7F, Supplementary Table S4).

**Figure 8:**
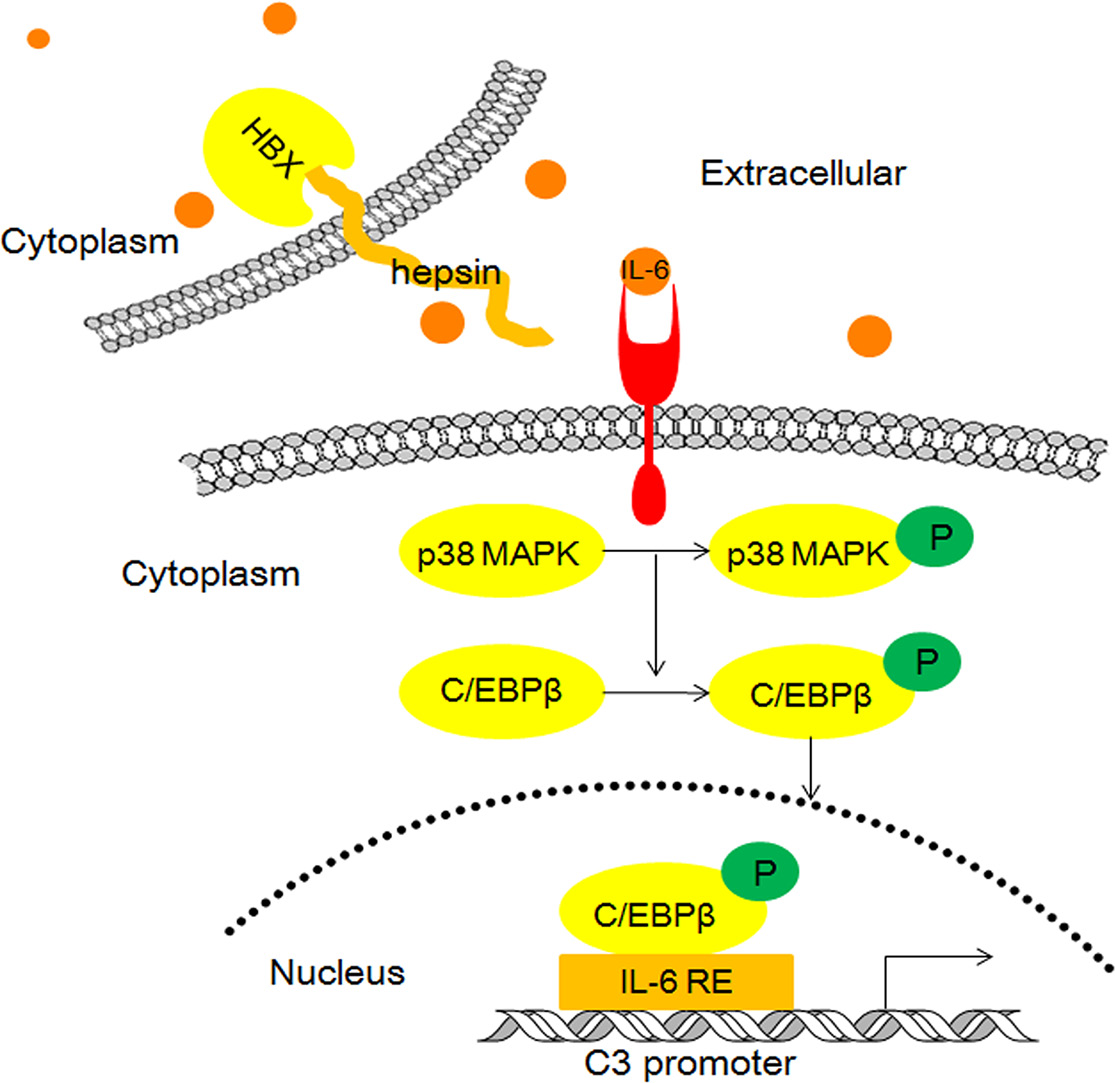
Model illustrating the possible mechanism of HBx and hepsin-induced C3 production in human hepatocytes

Based on these observations, we propose a model wherein HBx binding to hepsin promotes C3 production by inducing IL-6 secretion from hepatocytes (Figure 8).

## DISCUSSION

HBx acts via protein-protein interactions to play an important role in various signal transduction pathways related to cell apoptosis and carcinogenesis [33–35]. The interaction between HBx and hepsin appears to promote cell proliferation and block apoptosis in human liver tumor cells and normal liver cell lines alike [36]. Our study identified a novel mechanism in which HBx binding to hepsin induces C3 secretion from hepatocytes. Hepsin knockdown reduced C3 production. In clinical non-tumor liver tissues, hepsin levels were positively associated with C3 expression, which strongly suggests that hepsin is required for C3 production in normal liver. HBx also synergized with hepsin to up-regulate C3 mRNA levels and promoter activity in normal hepatocytes in which the p38 MAPK-C/EBP-β pathway was activated. By contrast, hepatitis C virus (HCV) NS5A and core protein down-regulate C3 mRNA levels and promoter activity by inhibiting the expression of C/EBP-β or the farnesoid X receptor [37].

The expression of C3 is regulated by IL-6, IL-1, TNF-α and glucocorticoids [29]. HBx and hepsin together enhanced pro-inflammatory IL-6 secretion, but had little or no effect on the secretion of IL-1β or TNF-α. Previous studies revealed that hepsin likely exhibits enzymatic activity toward the extracellular substrates pro-HGF [38, 39], pro-uPA [40], and laminin-332 [19]. It is therefore possible that altered signaling induced by hepsin-mediated cleavage and activation of macromolecular substrates may influence IL-6 secretion from hepatocytes.

HBx stimulates the synthesis and secretion of IL-6 through a MyD88-dependent pathway that involves the activation of both NF-κB and ERK/p38 MAPK in hepatic cells [41]. MyD88 is essential for HBx-stimulated IL-6 synthesis, and HBx transfection can increase mRNA and protein levels of MyD88. However, MyD88 does not interact directly with HBx [41]. Our study confirmed that increased IL-6 secretion resulting from the interaction of HBx and hepsin is not mediated by the regulation of hepsin expression, but by the direct binding of HBx and hepsin. Further study showed that HBx and hepsin cooperatively activated IL-6 mRNA expression and promoter activity by increasing the nuclear translocation of NF-κB. Within the nuclei, the p65 and p50 subunits of NF-κB may bind to the IL-6 promoter sequence, which is essential for the induction of the IL-6 gene [42]. IL-6 binds to its cognate receptor and activates the MAPK signaling pathway, resulting in activation of p38 MAPK. Phosphorylated p38 MAPK in turn phosphorylates and activates the transcription factor C/EBP-β, and subsequent binding to IL-6/IL-1 REs in the C3 promoter increases C3 promoter activity and gene expression (Figure 8).

Chronic HBV infection is a leading cause of progressive liver disease. Activation of the complement system has been shown to contribute to the protection of the host against viral infection [43]. Significant changes in C3, C4, and other components have been observed in patients acutely or chronically infected with HBV [6–9]. The increase or decrease of C3 appears to be based on the type and stage of hepatitis B infection. HBV is a hepatocyte-specific DNA virus that encodes four different viral proteins, including DNA polymerase, surface antigen, core antigen, and HBx [44, 45]. Various components of HBV may play different roles in modulating C3 expression. Here, we observed that the secretion of C3 was dramatically enhanced in flag-HBx-transfected hepatic cells. However, C3 secretion was weakly down-regulated in HBc-transfected LO2 cells and showed a tendency toward decrease in HBV-transfected liver cells. Similarly, HCV-encoded NS5A and core protein reduced C3 mRNA expression and promoter activity, whereas NS2 and NS3/4A had no effect on C3 mRNA expression and luciferase activity, further exemplifying the diverse effects of viral proteins on C3 production [37].

Our results offer a novel mechanism for complement activation resulting from the direct interaction of HBx and hepsin. HBx binding to hepsin protein enhances C3 promoter activation and expression by inducing IL-6 secretion from hepatocytes. Further studies will help to understand additional unique mechanisms of complement regulation by HBV proteins and identify targets with therapeutic potential.

## MATERIALS AND METHODS

### Cell culture and reagents

Human immortalized hepatic LO2 cells were maintained in Dulbecco's modified Eagle's medium (DMEM) supplemented with 10% fetal calf serum. CD1 mouse primary liver cells were maintained in appropriate culture media (Cell Biologics, USA) with 10% fetal calf serum. The specific p38 MAPK inhibitor SB203580 was from Beyotime (China). HBx recombinant protein was purchased from Prospec (USA).

### Clinical samples

Formaldehyde-fixed and paraffin-embedded clinical tumor or HBV-infected liver samples were immunohistochemically analyzed for hepsin, HBx, and C3 on tissue microarray slides (Shanghai Outdo Biotech Co, China). These samples included eleven human distant normal liver tissues, twelve pairs of human HCC and peripheral non-tumor tissues, four pairs of normal liver tissues and hemangioma, and three pairs of peripheral normal tissues and hepatocirrhosis. Out of 49 total samples, 35 liver tissue samples contained HBV, as confirmed by patients' serum positive HBsAg. Patients' consent and approval by the local ethics committee were obtained for the use of clinical materials in this research. Immunohistochemical staining was performed as described previously [15].

### ELISA

LO2 and mouse primary liver cells were cultured in 24-well plates. After transfection for 12, 24 and 48h, the supernatants were collected and C3 secretion was measured using commercially available ELISA kits (Abcam, UK). IL-6, IL-1β and TNF-α secretion were detected in the same way after transfection for 48h (RD, USA).

### Plasmid and siRNA transfections

The flag-tagged HBx and HBV vector was kindly provided by Dr. Michael Bouchard (Department of Biochemistry and Molecular Biology, Drexel University College of Medicine) [46]. V5-hepsin was made by cloning the entire human hepsin ORF into the pcDNA3.1 vector. Transient transfections were performed using Lipofectamine 2000 according to the manufacturer's instructions (Invitrogen, USA). Forty-eight hours after transfection, cellular mRNA and proteins were analyzed by real-time PCR and western blotting. Similarly, hepsin, IL-6 and C/EBP-β siRNAs synthesized by Genepharma (China) were transfected into LO2 and CD1 mouse primary liver cells with Lipofectamine 2000. After 72 hours, cells were lysed and cellular mRNA and proteins were analyzed by real-time PCR and western blotting. Culture supernatants were collected for C3 and IL-6 secretion assays.

### Real-time PCR

Cultured LO2 cells were transfected with the control vector, the HBx expression plasmid and the hepsin expression plasmid separately. The cells were harvested 48 hours after transfection, and total RNA was isolated using TRIzol reagent (Invitrogen, USA) according to the manufacturer's instruction. Total mRNA was converted to cDNA using AMV reverse transcriptase (Takara, Japan). Real-time PCR was carried out to amplify cDNA using SYBR Premix Ex Taq (Takara, Japan). C3 and IL-6 mRNA levels were evaluated using specific primers after normalization with glyceraldehyde3-phosphate dehydrogenase (GAPDH). The primer sequences were: C3, 5′-CAGCAGACCGCCCAGAGG-3′ (sense) and 5′-GGTCTGCCACACA-3′ (anti-sense); IL-6, 5′-ATGAACTCCTTCTCCACAA-3′ (sense) and 5′-CATTTGCCGAAGAGCCCT-3′ (anti-sense); GAPDH, 5′-GTCAAGGCTGAGAACGGGAA-3′ (sense), and 5′- AAATGAGCCCCAGCCTTCTC-3′ (anti-sense).

### Luciferase assay

Cells were transfected or treated with a promoter luciferase reporter construct (200 ng/well) alone or together with plasmids, siRNA or cytokines in a 24-well plate. The C3 promoter luciferase reporter construct was kindly provided by Dr. Ranjit Ray (Saint Louis University, USA). The IL-6 promoter luciferase reporter construct was purchased from Beyotime Biotechnology (China). Transfected cells were lysed using reporter lysis buffer (Promega, USA) 48 hours after transfection or 24 hours after cytokine treatment, and luciferase activity was measured using a Lumat LB9507 luminometer (EG&G Berthold, Bad Wildbad, Germany).

### Immunofluorescence

Cells were plated on coverslips overnight at 37°C and then fixed with 4% formaldehyde at room temperature for 15 min. The cells were treated with 0.2% Triton X-100 in PBS on ice for 10 min and then washed 4 times with 500μl PBS. HBx was detected with anti-HBx antibody (Abcam, UK) and visualized with goat anti-rabbit IgG-Alexa Fluor 594 (Jackson, USA). Hepsin was detected with anti-V5 antibody (Invitrogen, USA) and visualized with goat anti-mouse IgG-Alexa Fluor 488 (Jackson, USA). 4′, 6-diamidino-2-phenylindole dihydrochloride (DAPI) (Beyotime, China), was used for nuclear staining. Images were taken on a Confocal Laser Scanning Microscope (Leica TCS SP5, Germany).

### GST pull-down assay

GST and GST-hepsin were purified from bacterial lysates using glutathione-agarose beads (Amersham Biosciences). Purified GST-hepsin or control GST protein was incubated with HBx protein in binding buffer at 4°C overnight. Pre-equilibrated glutathione-Sepharose 4B beads were added and rotated overnight. Beads were then washed gently three times and boiled for 5 min, electrophoresed on a 10% SDS-PAGE, and analyzed by western blot.

### Preparation of cytoplasmic and nuclear fractions

Briefly, transfected cells were washed three times with ice-cold PBS and hypotonic buffer (10 mM HEPES [pH 7.9], 5 mM KCl, 1.5 mM MgCl_2_, 1 mM NaF, and 1 mM Na_3_VO_4_) supplemented with 1 mM DTT and 1 mM PMSF. After cells were lysed by incubating for 15 min on ice, the cytoplasmic fraction was prepared by centrifugation at 12,000g for 5 min. Following the initial centrifugation, the pellet was washed three times with hypotonic buffer and suspended in high-salt buffer (10 mM HEPES [pH 7.9], 1.5 mM MgCl_2_, 0.2 mM EDTA [pH 8.0], 420 mM NaCl, 25% glycerol, 50 mM β-glycerophosphate, 1 mM NaF, and 1 mM Na_3_VO_4_) supplemented with 1 mM DTT and 1 mM PMSF. The suspended cell pellet was incubated for 30 min on ice with occasional vortex, and the nuclear fraction was collected after centrifugation at 12,000g for 10 min.

### Immunoprecipitation

HEK293T cells were transfected with 4 μg of V5-hepsin and 4 μg of flag-HBx plasmids in 100-mm plates. Approximately 48 hours after transfection, cells were washed with ice-cold PBS and solubilized with immunoprecipitation buffer. Cell lysates were incubated with 2 μg of relevant antibody at 4°C for 2 hours. Pre-equilibrated protein G-agarose beads were added and incubated overnight, collected by centrifugation, and then gently washed three times with the lysis buffer. The bound proteins were eluted and analyzed using western blots.

### Western blotting

Total protein extracts or cytoplasmic and nuclear fractions were analyzed by SDS-PAGE and transferred onto a PVDF membrane (Roche Applied Science, Switzerland). Blots were blocked with PBS containing 5% non-fat dry milk and 0.1% Tween and then incubated with primary antibody. Primary antibodies were mouse anti-flag and anti-V5 antibodies (Invitrogen, USA); mouse anti-actin, rabbit anti-hepsin, and normal IgG antibodies (Santa Cruz Inc, USA); anti-phospho-p38, anti-phospho-C/EBP-β, anti-phospho-ERK, anti-phospho-JNK, anti-p38 anti-C/EBP-β, anti-ERK, anti-JNK, anti-p65, anti-p50, anti-histone H3 and anti-α-tublin (Cell Signal Technology, USA); anti-C3, anti-HBc and anti-HBx antibody (Abcam, UK).

### Statistical analysis

Experimental data were presented as means ±SEM of at least three independent replicates. Analyses were performed in GraphPad Prism 5, and groups were compared using a two-tailed Student's t-test. Differences in protein expression between paired HCC and non-tumor liver tissue samples were assessed by Wilcoxon ranks test in SPSS version 19.0. The correlation between hepsin/HBx and C3 staining obtained by immunohistochemistry was determined using Spearman's rank correlation test in SPSS version 19.0. *P < 0.05, **P < 0.01 and ***P < 0.001 were considered significant.

## SUPPLEMENTARY FIGURES AND TABLES


