# Supplementary material for: Hepatitis B virus X protein binding to hepsin promotes C3 production by inducing IL-6 secretion from hepatocytes
Source: Oncotarget. 2016 Jan 8;7(7):7780–800. doi: 10.18632/oncotarget.6846 (PMC4884954; doi:10.18632/oncotarget.6846)
Supplement: Supplementary file 1 [file oncotarget-07-7780-s001.pdf]

SUPPLEMENTARY FIGURES AND TABLES

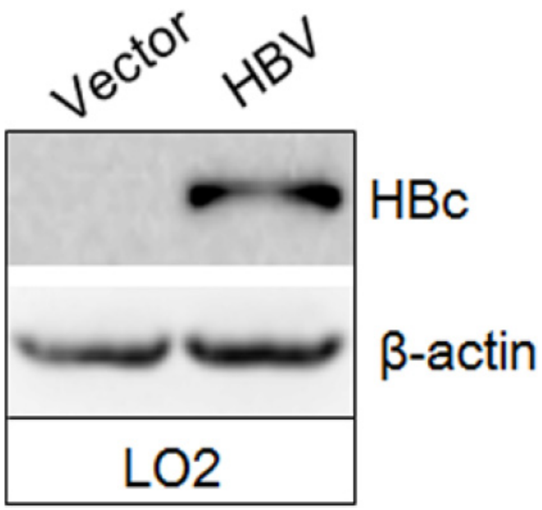

Supplementary Figure S1: LO2 cells were transfected with the HBV construct, core protein level was measured by western blot using anti-core antibody.

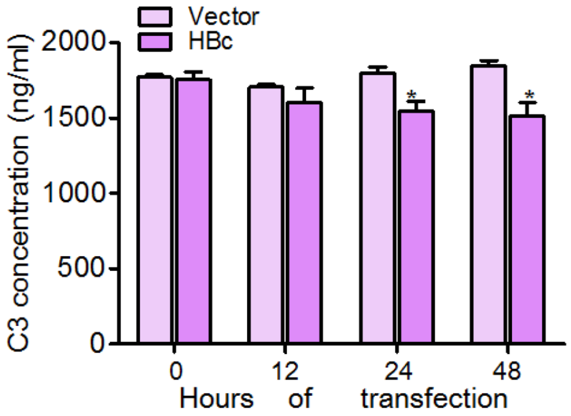

Supplementary Figure S2: HBc inhibits C3 secretion in LO2 cells. LO2 cells were transfected with HBc or empty vector. At indicated time points after transfection, C3 levels in the culture medium were measured by ELISA. Data are presented as mean ±SEM of three independent experiments. **P* < 0.05.

Supplementary Table S1: Comparison of hepsin expression in HCC with that in matched peripheral non-tumor tissues

Patient No.	Intensity of hepsin staining		*P* value
	HCC tissues	Matched peripheral non-tumor tissues	
1	0	1	
2	0	1	
3	0	2	
4	1	3	
5	1	2	
6	1	1	
7	1	2	0.013
8	1	2	
9	1	3	
10	1	1	
11	2	1	
12	2	3	

Note: Differences between variables were assessed by Paired Wilcoxon ranks test.

Supplementary Table S2: Comparison of C3 expression in HCC with that in matched peripheral non-tumor tissues

Patient No.	Intensity of C3 staining		*P* value
	HCC tissues	Matched peripheral non-tumor tissues	
1	1	3	
2	1	1	
3	1	2	
4	2	3	
5	2	1	
6	2	3	
7	2	3	0.166
8	2	1	
9	2	3	
10	2	3	
11	3	2	
12	3	3	

Note: Differences between variables were assessed by Paired Wilcoxon ranks test.

Supplementary Table S3: Correlation of hepsin expression in human non-tumor liver tissues with C3 expression

$P<0.05$ $r=0.449$

Intensity of C3 staining	Intensity of hepsin staining 1+	2+	3+
3+	2	3	3
2+	2	8	3
1+	3	2	0

Supplementary Table S4: Correlation of HBx expression in human HBV infected liver tissues with C3 expression

$P>0.05$ $r=0.168$

Intensity of C3 staining	Intensity of HBx staining 1+	2+	3+
3+	5	7	4
2+	3	5	6
1+	2	2	1

Note: The liver tissue contained HBV were confirmed by patients' serum HBsAg.
